# Cost-Effectiveness of ramucirumab plus paclitaxel as a second-line therapy for advanced gastric or gastro-oesophageal cancer in China

**DOI:** 10.1371/journal.pone.0232240

**Published:** 2020-05-07

**Authors:** Sini Li, Liubao Peng, Chongqing Tan, Xiaohui Zeng, Xiaomin Wan, Xia Luo, Lidan Yi, Jianhe Li

**Affiliations:** 1 The Xiangya Nursing School, Central South University, Changsha, Hunan, China; 2 Department of Pharmacy, The Second Xiangya Hospital, Central South University, Changsha, Hunan, China; 3 The Second Xiangya Hospital, PET-CT Center, Central South University, Changsha, Hunan, China; Universidade do Algarve Departamento de Ciencias Biomedicas e Medicina, PORTUGAL

## Abstract

**Aim:**

That clinical trial (RAINBOW) showed that a 7.4 months overall survival benefit with the combination therapy with ramucirumab (RAM) and paclitaxel (PAC) as second-line therapy for patients with recurrent or metastatic gastric or gastro-oesophageal junction adenocarcinoma, compared with placebo (PLA) plus paclitaxel. We performed an analysis to assess the cost-effectiveness of RAM from a Chinese perspective and recognized the range of drug costs.

**Methods:**

By building a Markov model to estimate quality-adjusted life-years (QALYs), life-years (LYs) and lifetime costs. Transition probabilities, costs and utilities were estimated for the published literature, Chinese health care system and local price setting. We performed threshold analyses and probabilistic sensitivity analyses to evaluate the uncertainty of the model.

**Results:**

Compared with PLA strategy, RAM strategy provided an incremental survival benefit of 1.22 LYs and 0.64 QALYs. The probabilistic sensitivity analysis showed that when RAM costs less than $151 or $753 per 4 weeks, the incremental cost-effectiveness ratio (ICER) approximated the willingness-to-pay threshold (WTP), suggesting that there was 50% likelihood that the ICER for RAM + PAC would be less than $44528.4 per QALY or $48121 per QALY, respectively.

**Conclusions:**

For patients with advanced gastric or gastro-oesophageal junction adenocarcinoma who fail first-line chemotherapy, our results are conducive to the multilateral drug price guidance negotiations of RAM in China.

## Introduction

Gastric cancer is the second most common malignancy in the world and the sixth leading cause of cancer mortality (8.2% of all the cancer deaths). [1, 2] According to valid statistics, each year, there are approximately 325,000 people die from gastric cancer in China. [3] Since 2012, China has had the highest incidence of new cases of gastric cancer in the world. [4] According to the latest reports, there were 7,872,000 new cases in 2018. [2] Pharmacoeconomic model studies have shown that the per capita disease-related expenditure of gastric cancer patients is about 30 thousand yuan per year, [5, 6] which is 12 times the per capita health expenditure in China, and that the annual expenditure is about 20 billion 370 million yuan.

Given the impetus to control the cost of gastric cancer treatment and accelerate the application of precision medicine in clinical practice, pharmacogenomics (PGx) has become increasingly important in the precision medicine of gastric cancer. [7] VEGF and VEGFR2 are associated with the pathogenesis and poor prognosis of gastric cancer. [8–10] The Current clinical application of VEGF monoclonal antibody such as trastuzumab and VEGFR2 antagonist such as RAM are benefit for patients with advanced gastric cancer and gastroesophageal cancer. [11, 12]

Currently, the chemotherapy, which base on fluoropyrimidine and platinum are the universally accepted first-line treatments for gastric cancer. [13] In 2010, the RAINBOW trial showed that, after failure of first-line chemotherapy, combination therapy with RAM and PAC, significantly increased overall survival (OS) and health-related quality of life (HR-QOL) for advanced gastric cancer patients who had been previously treated, compared to PAC strategy. [12, 14, 15] Therefore, the combination of RAM and PAC as a second-line treatment regimen for advanced gastric or gastro-oesophageal junction adenocarcinoma patients was included in the 2015 Chinese National Comprehensive Cancer Network (NCCN) Clinical Practice Guidelines in Oncology section on gastric cancer. [16]

Several studies have concurrently established that RAM has been approved in the United States, Europe and Japan, but it has not yet been approved by the China Food and Drug Administration (CFDA). [15, 17] The RAINBOW trial showed that RAM+PAC could enhance OS and PFS for patients with advanced gastric cancer. Among Asian patients (Region 3), the median OS [12.1 months (10.0–13.3)] and PFS [5.5 months (4.2–5.7)] were longer than in patients from other regions (Region 1 and Region 2). The median OS and PFS in Region 1 (USA, Europe, Australia, and Israel) and Region 2 (Argentina, Brazil, Chile, and Mexico) were similar, 8.5 months (7.4–9.8) and 4.2 months (3.4–4.9) respectively. Although RAM + PAC does prolong overall survival in gastric cancer patients, to date there has not been any pharmacoeconomic evaluation of this treatment and has no available information about the price of RAM in China mainland. [16] We therefore to carry out this medicine research deeply and expect that RAM will be approved in the future.

In Hong Kong, the price of RAM is $12.2/mg, but prices in the mainland are still undefined. Once RAM is approved by the CFDA, the drug will be use widely, and the out-of-pocket costs of cancer care will increase accordingly. Hence, we performed cost-effectiveness analysis and systematically varied drug costs to identify the range of drug costs from a Chinese perspective within which adding RAM as a second-line adjuvant regimen for advanced gastric cancer could be considered cost effective. This study can help the government in multilateral drug price guidance negotiations of RAM.

## Materials and methods

### Patients

The Markov model for the primary analysis was based on the RAINBOW trial, a randomized, placebo-controlled, double-blind, phase 3 trial that compared RAM + PAC with PLA + PAC as second-line therapy for patients with metastatic or non-resectable advanced gastric or gastro-oesophageal junction adenocarcinoma. [12] This trial was conducted at 170 study sites across 27 countries. Briefly, the trial enrolled 665 patients with stage IV gastric cancer who were 18 years or older, had disease progression or after 4 months first-line drug therapy (platinum and fluoropyrimidine doublet with or without anthracycline) failed, and had eligibility criteria included an performance status score of 0 or 1 in Eastern Cooperative Oncology Group (ECOG). [12] Patients were allocated to two strategies in a 1:1 ratio randomly and stratified by three geographic regions.

### Treatment

The 330 patients in the RAM group received RAM (8 mg/kg intravenously on days 1 and 15 of a 28-day cycle) and PAC (80 mg/m^2^ intravenously on days 1, 8 and 15 of a 28-day cycle). The trial assessed the quality of life every 6 weeks until disease progression. Radiological examinations (such as CT scans) were performed every 6 weeks. Treatment was administered at the beginning of each cycle. Progression-free survival was assessed at each cycle. At the beginning and end of each treatment cycle or the end of the 30-day follow-up, the functional status of patients was assessed.

### Economic model

**Fig 1:**
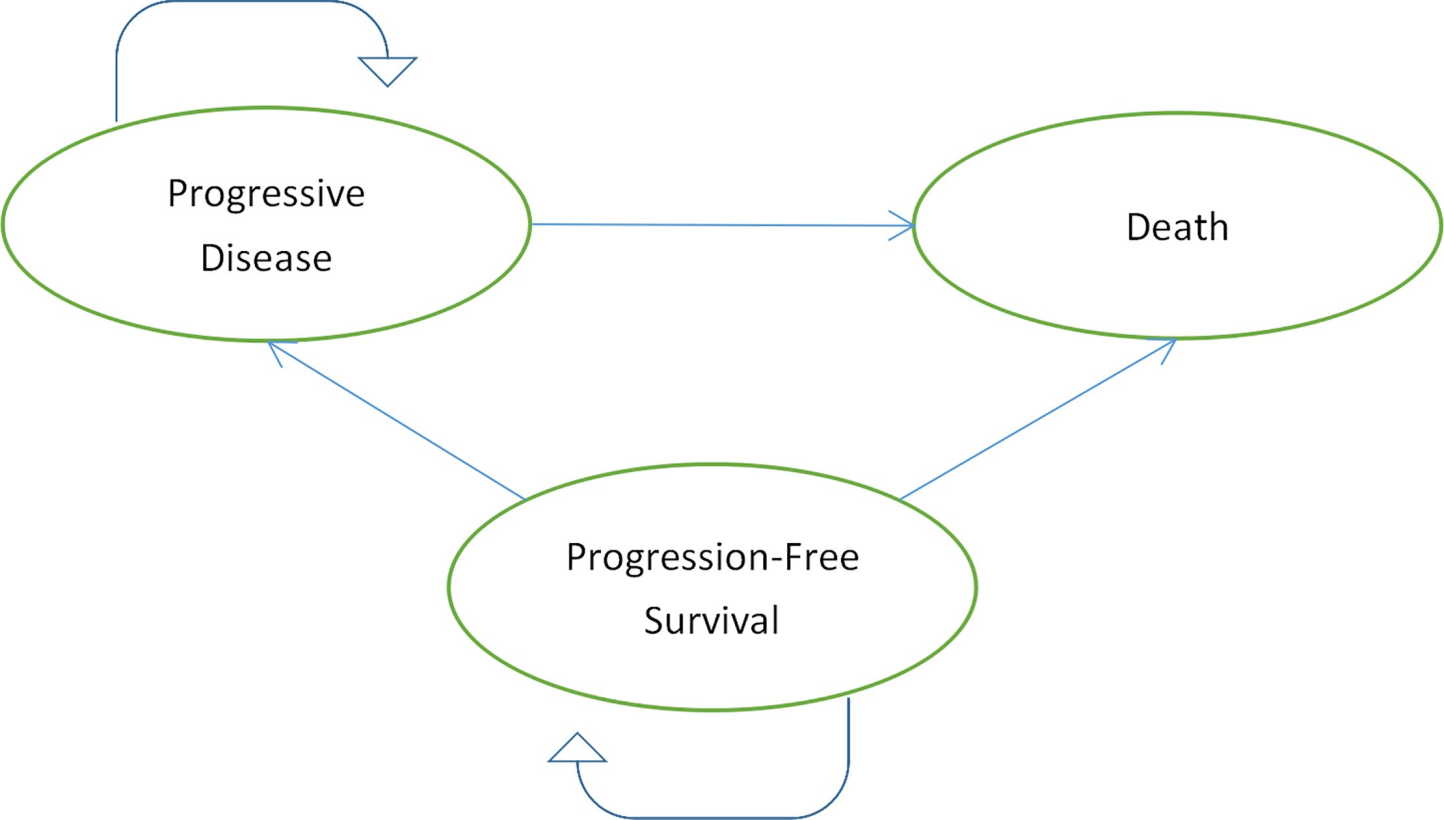
Markov model for patients with advanced gastric or gastro-oesophageal cancer.

Patients were classified as being in one of three states: progression-free survival (PFS), progressive disease (PD) or death.(Fig 1) We used Getdata Graph Digitizer (version 2.25; http://www.getdata-graph-digitizer.com/index.php) to extract the probability of being in each state based on the PFS and OS as reported in the published Kaplan-Meier curves from the RAINBOW trial. Based on Eq 1 and Eq 2 below, we were assessed the transition probabilities of each health states.

Time-dependent transition probabilities from PFS to PD:

Tp(tU)=(1+exp(Theta)*(u)^(Kappa))/(1+exp(Theta)*(u+1)^(Kappa)) (Eq 1)

Time-dependent transition probabilities from PFS or PD to death:

Tp(tU)=1‐(1+exp(Theta)*(u)^(Kappa))/(1+exp(Theta)*(u+1)^(Kappa)) (Eq 2)

where u denotes the Markov cycle and t_u_ manifests that t is calculated as integer multiples of the period length of the model. [18]

We used R-studio software (http://www.r-project.org) to fit the log-logistic survival curve of the PLA group, and then used the hazard rate to generate the survival curve of the RAM group. Using TreeAge Pro2018 software (TreeAge, Williamstown, MA), and then by estimating a Markov model to model the treatment sequences among advanced gastric cancer and a value-based cost in China was established. The estimated parameters (Theta and Kappa) are listed in Table 1.

**Table 1:** Log-logistic parameters for Progressive-Free Survival (PFS) and Overall Survival (OS) for two strategies.

	Theta	Kappa
**PFS**		
Ramucirumab	-3.157	2.058
placebo	-2.268	2.042
**OS**		
Ramucirumab	-4.671	2.089
placebo	-3.337	1.681

Patients in group 1 received timely treatment with RAM and PAC. Patients in group 2 received timely treatment with PLA and PAC. (Fig 1) The model cycle length was 28 days because ramucirumab was administered every 28 days. And model simulation runs for 5 years. A partition survival model was built to estimate costs, LYs, and QALYs.

### Costs and utilities

The direct medical costs were considered from the Chinese perspective of the health care system, including costs of the drugs and optimal supportive treatment, administration, management of adverse reactions and follow-up. Treatment was administered in every cycle, and the costs of administration were based on the study by Chongqing T et al. [6] We assumed that patients in the current study weighed 65 kg [6] and should be administered 520 mg of RAM. The cost of PAC per 30 mg was obtained from the local health system. [19] The cost of grade 3/4 adverse effects (AEs) included haematotoxicity, hypertension, fatigue and abdominal pain were obtained from our previously published research or other published economic evaluation. [6, 20]The incidence rates of adverse events in the model were taken from the RAINBOW trial.All direct costs were listed in Table 2.

**Table 2:** Model economic parameters and the range of the sensitivity analysis.

Variable	Median	Range	Distribution
**Treatment costs, $**			
Paclitaxel/30mg [19]	116.2	58.1 to 174.3	Log-normal
Administration per unit [6]	18.6	16.6 to 23.2	Log-normal
Local laboratory per unit [21]	88.2	35.4 to 184.0	Log-normal
Best supportive care per unit [19]	1425.1	1029.8 to 2035.4	Log-normal
Abdominal CT per unit [6]	105.9	53.0 to 158.9	Log-normal
**Costs Serious adverse events, $**			
Decreased appetite and fatigue per episode [6]	116.2	104.5 to 127.8	Log-normal
Abdominal pain and diarrhoea per episode [6]	44.6	28.7 to 55.0	Log-normal
Nausea and vomiting per episode [6]	40.0	18 to 77	Log-normal
Neutropenia per episode [6]	534.4	199.9 to 869.0	Log-normal
Hypertensions [20]	16.6	14.9 to 18.15	Log-normal
**Risks of serious adverse events in ramucirumab group(grade 3 or 4)**^b^			
Decreased appetite and fatigue [12]	0.15	0.12 to 0.18	Beta
Abdominal pain and diarrhoea [12]	0.098	0.0784 to 0.1176	Beta
Nausea and vomiting [12]	0.043	0.0344 to 0.0516	Beta
Neutropenia [12]	0.41	0.328 to 0.492	Beta
Hypertension [12]	0.14	0.112 to 0.168	Beta
**Risks of serious adverse events in placebo group(grade 3 or 4)**			
Decreased appetite and fatigue [12]	0.094	0.0752 to 0.1128	Beta
Abdominal pain and diarrhoea [12]	0.043	0.0344 to 0.0516	Beta
Nausea and vomiting [12]	0.061	0.0488 to 0.0732	Beta
Neutropenia [12]	0.19	0.152 to 0.228	Beta
Hypertension [12]	0.02	0.016 to 0.024	Beta
**Discount rate,% [22]**	3	0 to 8	Fixed in PAS
**Utility**			
Progressed disease [26]	0.42	0.28 to 0.63	Beta
Progression-free survival [26]	0.68	0.61 to 0.75	Beta
**Weight, kg**^a^^,^ **[6]**	65	52.0 to 78.0	Log-normal
**Body surface area,m**^**2,**^^b^^,^ **[23]**	1.62	1.296 to 2.43	Log-normal

^a^ The range was varied by ±50%

^b^ The range was varied by ±20%

All costs from past sources were adjusted to 2018 US dollars (USD) at a rate of 1 USD to 6.889 RMB and adjusted to 2018 dollar values with use of the Bureau of Statistics Consumer Price Index of China. [24] We adopted a 3% discount rate per year for costs and QALYs. [22] The World Health Organization (WHO) guidelines suggested that using 1.5–3 times the per capita gross domestic product (GDP) as the WTP threshold. Using 1.5 times and 3 times the per capita GDP of China 2017 were $13010.95 and $26021.9. Using 1.5 times and 3 times the per capita GDP of Beijing city 2017 were $28131.65 and $56263.3. [16, 25] Because this study considered hospice costs, in accordance with TreeAge Pro 2018 instructions, a half-cycle correction of health status and terminal cost was implemented.

In RAINBOW trial, they using the European Organisation for Research and Treatment of Cancer quality-of-life questionnaire (EORTC QLQ-C30, version 3.0) and the EuroQoL five-dimension, three level health status questionnaire (EQ-5D-3L) to assess quality of life. However, there is no information about the utility scores of PFS and PD in the RAINBOW trial, we derived the values from previously published evaluation. The utility for the state of progression-free survival, progressed disease and death were assumed at 0.68, 0.42 and 0, respectively, on the basis of a report by Lam SW et al. [26]

### Sensitivity analyses

In this research we do the one-way sensitivity analyses and probability sensitivity analyses (PSA). One-way sensitivity analyses were conducted to identify the parameters that most significantly influenced the economic outcomes. The PSA was used to obtain the robustness of the model and explore the uncertainty of the variable estimation. The model was run 1000 times, in which the parameters were changed with a specific pattern of distribution simultaneously. For these analyses, we set a value range for all the parameters to assess the effect of the lower and upper bound values on the final results. The mean value of each distribution was presumed to be their baseline values, furthermore, the standard error was accordingly set to 10% of baseline values. The alternative distributions considered included lognormal and Beta distribution. Lognormal distribution was chosen for all the direct cost. Beta distribution was chosen for the risks of serious adverse events. The distributions used in probabilistic sensitivity analyses (PSA) and one-way analysis are summarized in Table 2. All variables of cost and the risks of ADs varied over a plausible range (Table 2), which were obtained from credible intervals or by assuming ±50% and ±20%, respectively, of base case values. From Chinese perspective, Curves which represents the probability that the ICER is below the willingness-to-pay (WTP) thresholds for different costs of RAM were presented. To do the PSA, with adjusted the price of RAM constantly, the model was run 1000 times per adjustment through the software and all of variables were changed a thousand times in its range, to get the probability of cost-effectiveness of ramucirumab at two different WTP. When we set the cost of RAM fix the x-axis at a certain value, the figure showed that two curves for the cost effectiveness of RAM at a fixed price. (Fig 3)

### Changes in the cost of drug

We determine the impact on the ICER by running the base case model multiple times with different costs of RAM, which resulted in WTP beyond the ICERs were presented.

## Results

### Base case results

In the base case analysis showed that over 5-year time horizon, the model reflected that the life expectancy of patients receiving RAM + PAC provided 1.22 LYs or 0.64 QALYs, which was gained an extra 0.04 LYs or 0.07 QALYs, compared with those receiving PLA + PAC. When RAM cost $244 and $604 per 4weeks, the RAM + PAC group the incremental cost-effectiveness ratio (ICER) was $26014 per QALY or $56260 per QALY, respectively. (Table 3)

**Table 3:** The base-case analysis between mainland China and Beijing City.

	Base Case Analysis	
Parameter	Mainland China	Beijing
WTP value, $/QALY	26022	56263
Ramucirumab cost, $/4 weeks	244	604
Total cost, $	45081.36	47231.66
Lys	1.22	1.22
QALYs	0.64	0.64
ICER, $/LY	16810	18961
ICER, $/QALY	26014	56260

Abbreviations: WTP, Willingness-to-pay; QALY, quality-adjusted life-year; ICER, incremental cost-effectiveness ratio; LY, life-year.

### Sensitivity analyses

**Fig 2:**
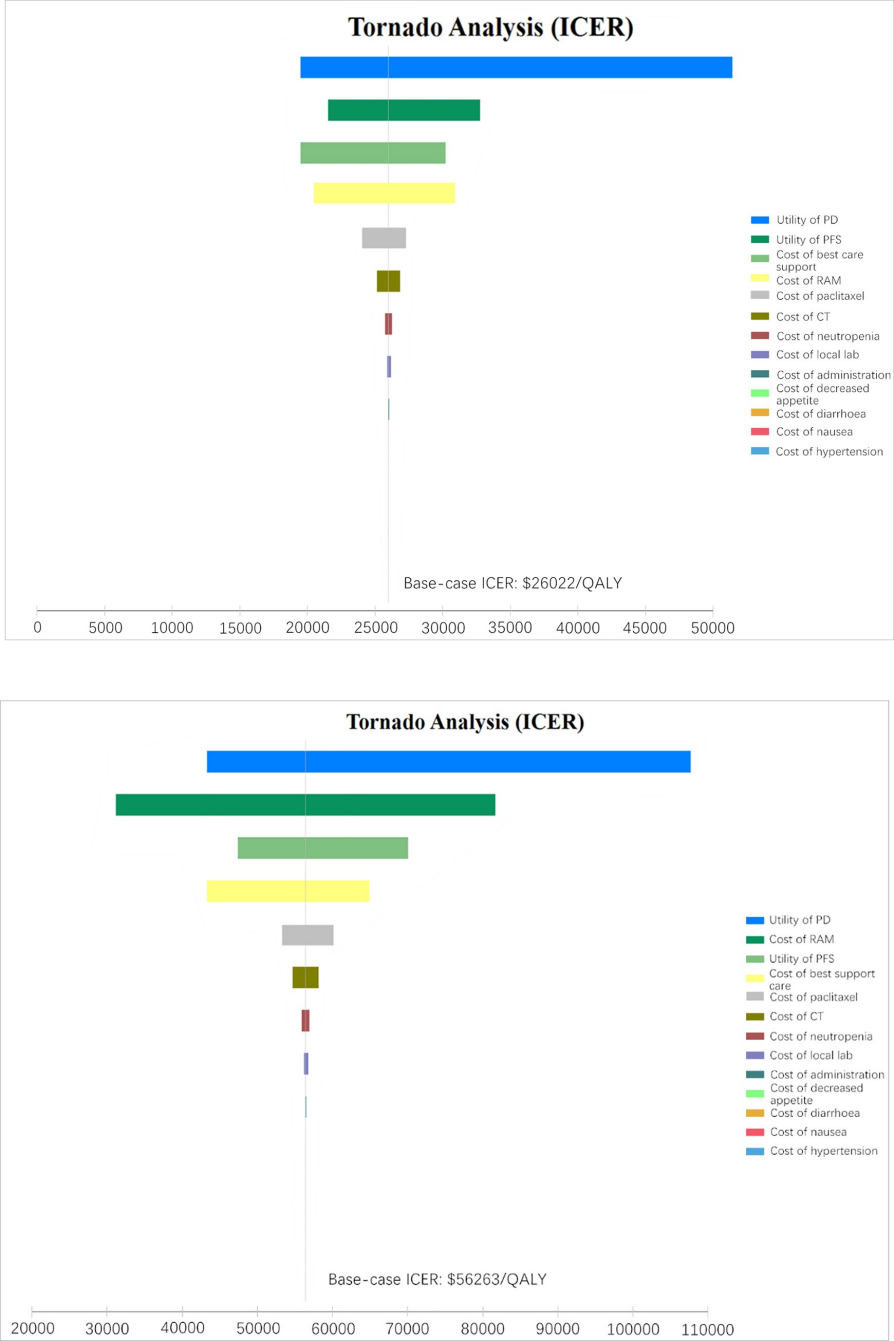
Tornado diagram of the one-way sensitivity analysis of the Incremental Cost-Effectiveness Ratio (ICER) of RAM over chemotherapy in all patients in the mainland China (A) and the Beijing city (B). PD, progressed disease; PFS, Progressed-free disease; RAM, ramucirumab.

**Fig 3:**
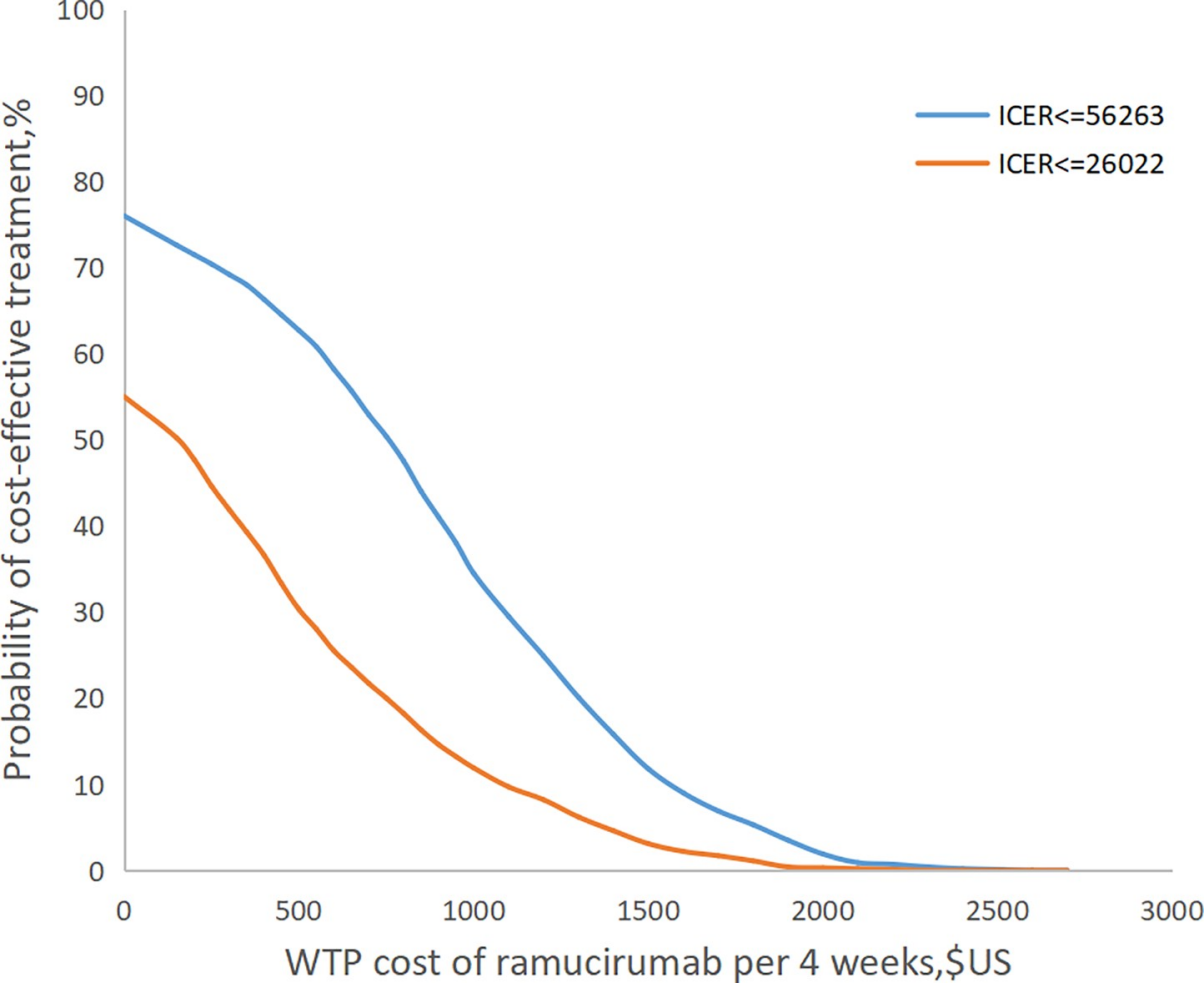
Ramucirumab cost-effectiveness curves under different costs.

Based on Table 3, we performed a one-way sensitivity analysis. (Fig 2) In this study, WTP in mainland China mainland ($26022/QALY) and Beijing city ($56263/QALY) was used as the baseline values for tornado diagram. The model outcome was sensitive to the utility of progressed disease (PD), utility of progressed-free disease (PFS) and cost of best support care in the China mainland and was sensitivity to the utility of PD, cost of RAM and utility of PFS in the Beijing city. Other variables, such as the cost of paclitaxel, cost of CT, cost of adverse drug event (ADR) and cost of administration, had a moderate or mild impact on the economic outcomes. The results of probability sensitivity analyses for China or for Beijing city, Shown in Fig 3, suggest that when RAM was priced at $0 per 4week, the probability that RAM would be cost-effective was 55% or 76%, respectively. When the price of RAM is less than $600 or $1200 per 4 weeks, there was a nearly 75% probability that the RAM would be no cost-effectiveness in China mainland or Beijing city. When the RAM cost is greater than $2600 per 4 weeks, the probability of the ICERs exceeding WTP thresholds in China is 100%.

## Discussion

We varied the cost of RAM using a probabilistic sensitivity analysis with 1,000 Monte Carlo simulations. When the price of RAM was set at $0.2343/mg (RAM cost less than $244 per 4 weeks) in China, the ICER ($26014/QALY) approximated the WTP threshold ($26022/QALY), and the RAM+PAC combination was cost-effective in 48.3% of simulations. When the price of RAM was set at $0.5808/mg (RAM cost less than $56260 per 4 weeks) in Beijing, the ICER ($56260/QALY) approximated the Beijing-specific WTP threshold ($56263/QALY), and the RAM+PAC combination was cost-effective in 50.9% of simulations, suggesting that this treatment would be a cost-effective option in Beijing City. One cost-effectiveness analysis from the US demonstrated that the combination of RAM and PAC was not cost-effective (when the ICER greater than $50,000). [26] The probabilistic sensitivity analyses in that study showed that when the WTP threshold less than $400,000/QALY, the RAM + PAC combination was not cost-effective. [26] Compared with other second-line adjuvant regimens, RAM + PAC resulted in the highest QALY gains, but at a cost that exceeds the WTP threshold. [26] Actually, for patients with advanced cancer disease whose survival is limited, not only the RAM, but many other cancer medicines are not cost-effective due to their low incremental benefits and high incremental costs. [27] In US, many low-value drugs such as regorafenib, bevacizumab and pertuzumab are covered in Medicare. [28–30] For low-value drugs in US, there are few barriers to coverage because Medicare must reimburse any Food and Drug Administration (FDA) approved drugs regardless of price. [31] However, in China, many new cancer drugs are not covered by National Reimbursement Drug List, for Chinese patients who suffer financial toxicity greatly are pay out of pocket. The cost of cancer treatment has been rising rapidly around the world, and the introduction of expensive new anticancer medicines has leaded to this phenomenon. [32] Recently, according to the latest version of the Regulations on the Import and Export Tariffs of the People's Republic of China, the import tariff rate will decrease to zero on May 1, 2018. The new regulations will cover medicines including cancer drugs, ordinary drugs, alkaloids with anticancer activity and traditional Chinese medicines. With the elimination of tariffs on drugs, the prices of many imported anti-cancer chemotherapy drugs will decrease.

The worldwide use of adjuvant chemotherapy has come at great financial costs. Ignoring interruptions and dose reductions, the cost of the RAM + PAC combination was US$ 191,776 [(65 kg [6]×2×8×C1+1.67 m^2^ [23]×3×C2)×13, where 1.67 is the body surface area, C1 is US$ 12.2 (the cost of RAM per 1 mg in Hongkong), C2 is US$ 154.6 (the cost of PAC per 30 mg)] for 364 days (thirteen 28-day cycles) for one patient. Achieving the appropriate balance of treatment benefits and costs will require decreasing the price of the drug or taking advantage the potential economic impact of new chemotherapy regimens.

These results can help local governments to consider RAM treatment after failure of first-line chemotherapy for locally advanced gastric cancer in accordance with the level of local economic development. There are several limitations in our study that warrant discussion. First, the utility values used for Chinese patients with advanced gastric cancer were obtained from Western countries, because there are still no utility data in China, and the theoretical value that we estimate here may differ from the true value in clinical practice. However, the sensitivity analysis revealed that there will not be a significant influence on the cost-effectiveness analysis results. Second, we only considered grade 3/4 AEs in the model. We hypothesized that low-probability adverse events would not change the final findings of the study, because the sensitivity analysis showed that the result was not sensitive to changes related to AEs. Third, some other expenses such as the costs of travel, lodging, additional imaging, and time missed from work due to the disease were not considered. Finally, we did not evaluate the affect and costs of various treatments after disease progression. We built the Markov model based on NCCN guidelines and RAINBOW trial, which may not reflect the current Chinese clinical practice situation precisely. According to previous research, because most of oncologists using NCCN guidelines to make the clinical decisions, the difference in clinical practice for advanced gastric cancer between China and the United states has little impact on the conclusions of our research. [31] Furthermore, the cost data in the model were derived from previously published Chinese-based literature. And the Chinese-value-based price of ramucirumab in this research is contribute to multilateral drug price negotiations of the cost and value of cancer treatment. With the sharp rise of drug prices, imperfect insurance coverage and high out-of-pocket costs in China, cost-benefit analysis plays an important role in the decision-making and pricing of insurance coverage. Meanwhile, the results of sensitivity analyses with a wide range of variation showed that the results were stable. We believe that our results provide useful references in guiding decisions regarding the price of RAM in China.

## Conclusion

Our results show that for Chinses patients with advanced gastric cancer, second-line adjuvant therapy with RAM + PAC is unlikely to be cost-effective for reasonable and expected ranges of drug cost. Efforts to develop adjuvant chemotherapy that increases QOL and decrease the price of RAM would be better options to meet the needs of China and Chinese patients.

## Supporting information

S1 Checklist STROBE statement—Checklist of items that should be included in reports of observational studies. (DOCX)
