# Supplementary material for: Cost-Effectiveness of ramucirumab plus paclitaxel as a second-line therapy for advanced gastric or gastro-oesophageal cancer in China
Source: PLoS One. 2020 May 7;15(5):e0232240. doi: 10.1371/journal.pone.0232240 (PMC7205241; doi:10.1371/journal.pone.0232240)
Supplement: S1 Checklist — (DOCX) [file pone.0232240.s001.docx]

STROBE Statement—checklist of items that should be included in reports of observational studies

|  | Item No | Recommendation |
| --- | --- | --- |
| **Title and abstract** | 1-2 | (a)Cost-Effectiveness of ramucirumab plus paclitaxel as a second-line therapy for advanced gastric or gastro-oesophageal cancer in China. |
|  |  | (b) By building a Markov model to estimate and compare quality-adjusted life-years (QALYs), life-years (LYs) and lifetime costs of Ramucirmab (RAM) plus paclitaxel and placebo (PLA) plus paclitaxel. Compared with PLA strategy, RAM strategy provided an incremental survival benefit of 1.22 LYs and 0.64 QALYs. For patients with advanced gastric or gastro-oesophageal junction adenocarcinoma who fail ﬁrst-line chemotherapy, our results are conducive to the multilateral drug price guidance negotiations of RAM in China. |
| Introduction | | |
| Background/rationale | 2-3 | According to the latest reports, there were 7,872,000 new cases in 2018. Pharmacoeconomic model studies have shown that the per capita disease-related expenditure of gastric cancer patients is about 30 thousand yuan per year, which is 12 times the per capita health expenditure in China, and that the annual expenditure is about 20 billion 370 million yuan.Currently, the chemotherapy, which base on fluoropyrimidine and platinum are the universally accepted first-line treatments for gastric cancer. In 2010, the RAINBOW trial showed that, after failure of first-line chemotherapy, combination therapy with RAM and PAC, significantly increased overall survival (OS) and health-related quality of life (HR-QOL) for advanced gastric cancer patients who had been previously treated, compared to PAC strategy. |
| Objectives | 4 | Although RAM + PAC does prolong overall survival in gastric cancer patients, to date there has not been any pharmacoeconomic evaluation of this treatment and has no available information about the price of RAM in China mainland. We therefore to carry out this medicine research deeply and expect that RAM will be approved in the future. |
| Methods | | |
| Participants | 4 | The RAINBOW trial enrolled 665 patients with stage IV gastric cancer who were 18 years or older, had disease progression or after 4 months first-line drug therapy (platinum and fluoropyrimidine doublet with or without anthracycline) failed, and had eligibility criteria included an performance status score of 0 or 1 in Eastern Cooperative Oncology Group (ECOG).[12] Patients were allocated to two strategies in a 1:1 ratio randomly and stratified by three geographic regions. |
| Setting | 4-5 | The Markov model for the primary analysis was based on the RAINBOW trial, a randomized, placebo-controlled, double-blind, phase 3 trial that compared RAM + PAC with PLA + PAC as second-line therapy for patients with metastatic or non-resectable advanced gastric or gastro-oesophageal junction adenocarcinoma. This trial was enrolled 665 patients and conducted at 170 study sites across 27 countries. |
| Treatment | 5 | The 330 patients in the RAM group received RAM (8 mg/kg intravenously on days 1 and 15 of a 28-day cycle) and PAC (80 mg/m2 intravenously on days 1, 8 and 15 of a 28-day cycle). The trial assessed the quality of life every 6 weeks until disease progression. Radiological examinations (such as CT scans) were performed every 6 weeks. Treatment was administered at the beginning of each cycle. Progression-free survival was assessed at each cycle. At the beginning and end of each treatment cycle or the end of the 30-day follow-up, the functional status of patients was assessed. |
| Study design | 5-6 | Using TreeAge Pro2018 software (TreeAge, Williamstown, MA), and then by estimating Markov model to model the treatment sequences among advanced gastric cancer and a value-based cost in China was established. |
| Statistical methods | 5-6 | We used Getdata Graph Digitizer (version 2.25; http://www.getdata-graph-digitizer.com/index.php) to extract the probability of being in each state based on the PFS and OS as reported in the published Kaplan-Meier curves from the RAINBOW trial. We used R-studio software (<http://www.r-project.org>) to fit the log-logistic survival curve of the PLA group, and then used the hazard rate to generate the survival curve of the RAM group. Using TreeAge Pro2018 software (TreeAge, Williamstown, MA), and then by estimating a partitioned survival model to model the treatment sequences among advanced gastric cancer and a value-based cost in China was established. |
| Variables | 7-8 | The direct medical costs were considered from the Chinese perspective of the health care system, including costs of the drugs and optimal supportive treatment, administration, management of adverse reactions and follow-up. And we also use utility values to calculate the status of disease. |
| Data sources/ measurement | 7-9 | The direct cost obtained from local drug store and/or published articles.  The utility for the state of progression-free survival, progressed disease and death were assumed at 0.68, 0.42 and 0, respectively, on the basis of a report by Lam SW et al. |
| Sensitivity analyses | 9 | In this research we do the one-way sensitivity analyses and probability sensitivity analyses (PSA). |

Continued on next page

| Results | | |
| --- | --- | --- |
| Base case result | 11 | In the base case analysis showed that over 5-year time horizon, the model reflected that the life expectancy of patients receiving RAM + PAC provided 1.22 LYs or 0.64 QALYs, which was gained an extra 0.04 LYs or 0.07 QALYs, compared with those receiving PLA + PAC. When RAM cost $244 and $604 per 4weeks, the RAM + PAC group the incremental cost-effectiveness ratio (ICER) was $26014 per QALY or $56260 per QALY, respectively. |
| Sensitivity analyses | 11-12 | The model outcome was sensitive to the utility of progressed disease (PD), utility of progressed-free disease (PFS) and cost of best support care in the China mainland and was sensitivity to the utility of PD, cost of RAM and utility of PFS in the Beijing city. |
| Main results | 12 | when RAM was priced at $0 per 4week, the probability that RAM would be cost-effective was 55% or 76%, respectively. When the price of RAM is less than $600 or $1200 per 4 weeks, there was a nearly 75% probability that the RAM would be no cost-effectiveness in China mainland or Beijing city. When the RAM cost is greater than $2600 per 4 weeks, the probability of the ICERs exceeding WTP thresholds in China is 100%. |
| Discussion | | |
| Key results | 12 | Efforts to develop adjuvant chemotherapy that increases QOL and decrease the price of RAM would be better options to meet the needs of China and Chinese patients. |
| Limitations | 14 | First, the utility values used for Chinese patients with advanced gastric cancer were obtained from Western countries, because there are still no utility data in China, and the theoretical value that we estimate here may differ from the true value in clinical practice.Second, we only considered grade 3/4 AEs in the model.Third, some other expenses such as the costs of travel, lodging, additional imaging, and time missed from work due to the disease were not considered. Finally, we did not evaluate the affect and costs of various treatments after disease progression. |
| Interpretation | 15 | Our results show that for Chinses patients with advanced gastric cancer, second-line adjuvant therapy with RAM + PAC is unlikely to be cost-effective for reasonable and expected ranges of drug cost. Efforts to develop adjuvant chemotherapy that increases QOL and decrease the price of RAM would be better options to meet the needs of China and Chinese patients. |
| Other information | | |
| Funding |  | No |
